# Test and Microstructural Analysis of a Steel Slag Cement-Based Material Using the Response Surface Method

**DOI:** 10.3390/ma15093114

**Published:** 2022-04-25

**Authors:** Xuanshuo Zhang, Hongbo Li, Sheng Li, Yongfa Ding, Hubiao Zhang, Yufei Tong, Shudong Hua

**Affiliations:** 1 College of Civil and Hydraulic Engineering, Ningxia University, Yinchuan 750021, China; zxsnikea@163.com (X.Z.); 13995891649@sina.cn (S.L.); nxumrding@163.com (Y.D.); zhanghubiao1222@163.com (H.Z.); tongyufei1028@163.com (Y.T.); 2 Ningxia Research Center of Technology on Water-Saving Irrigation and Water Resources Regulation, Yinchuan 750021, China; 3 Engineering Research Center for Efficient Utilization of Water Resources in Modern Agriculture in Arid Regions, Yinchuan 750021, China; 4 Ningxia Huasheng Energy Saving and Environmental Protection Technology Co., Ltd., Yinchuan 750021, China; ytmn0420@gmail.com

**Keywords:** steel slag cement-based material, response surface method, optimal preparation parameters, interaction, interface bonding strength

## Abstract

In this study, the silica fume replacement rate, fly ash replacement rate, and curing temperature were regarded as the independent variables, and the compressive and flexural strengths were regarded as the response values. The response surface method was used to construct the response surface polynomial regression model and obtain the optimal preparation parameters of a steel slag cement-based gel slurry (SCGS). The univariate and multivariate effects on the SCGS’s strength were investigated via analysis of variance and a three-dimensional surface model, and the hydration products and strength development law were characterized via scanning electron microscopy and X-ray diffraction. The actual compressive strengths at 3 and 28 d of age were 31.78 and 53.94 MPa, respectively, which were close to the predicted values (32.59 and 55.81 MPa, respectively), demonstrating that the optimized strengths were accurate and reliable. Further, the hydration reaction rate of SiO_2_ in the silica fume and the physical filling effect of the inert components of fly ash and steel slag under the optimal parameters were the key factors for the early strength of the material. Moreover, continuous C_3_S hydration in steel slag and the continuous excitation of the volcanic ash properties of fly ash were important factors for the later strength.

## 1. Introduction

For many years, China has been among the major steel-producing countries in the world [1]. Steel slag is the main industrial waste in the steelmaking process; in 2020 alone, the output reached 161 million tons, but the utilization rate was only 30%. The low utilization rate of steel slag causes its accumulation, which is detrimental to environmental protection [2,3]; hence, research on the comprehensive utilization of steel slag is necessary. In large-scale infrastructure, Portland cement is the main building material, and its production requires large amounts of coal and limestone; moreover, its emissions include harmful gases, such as CO_2_ and SO_2_ [4]. Many scholars have proposed steel slag as a mineral admixture in the production of cement concrete by replacing part of the cement [5,6]; this can not only solve many environmental problems caused by the massive accumulation of local steel slag but can also contribute to the achievement of carbon neutralization goal [7,8]. However, when steel slag is used as a mineral admixture, the active minerals, namely, dicalcium silicate (C_2_S) and tricalcium silicate (C_3_S), are not fully hydrated and a high content of free calcium oxide (f-CaO) leads to the poor volume stability of concrete, resulting in the poor working performance and limited durability of a steel slag cement system [9]. Therefore, making full use of steel slag requires stimulating its activity and solving the problem of stability.

At present, the mechanical activation method and active fine admixture compound method have been widely used to stimulate the activity of steel slag. In particular, mechanical grinding increases the specific surface area of steel slag and hydrates it fully. Furthermore, the Ca(OH)_2_ produced during the f-CaO hydration in steel slag is consumed by active SiO_2_ and Al_2_O_3_ in a fine admixture, thereby hindering the concrete expansion caused by f-CaO [10]. The synergistic effect of fine admixture facilitates the production of high-performance concrete, the effective utilization of industrial waste, the development of strengths, and the elimination of weaknesses [11,12]. Several scholars have studied the mix proportion design of composite cementitious materials prepared by replacing part of the cement with fine mineral admixtures [13,14,15], the microstructure [16], the hydration mechanism [17], the working performance [18], etc. Zhang et al. [19] attained strong mechanical properties of concrete by compounding steel slag powder and fly ash in a 2:1 ratio; moreover, the carbonation resistance and chloride ion penetration resistance are significantly improved [20]. Wang et al. [21] mixed steel slag and furnace slag in a 3:7 ratio to replace 50% of cement to prepare concrete, demonstrating that the strength and hydration heat can be significantly improved. The addition of a fine active mineral admixture can subject the cement to secondary hydration, reduce Ca(OH)_2_, promote the gel formation of calcium silicate hydrate (C–S–H) and calcium aluminate hydrate (C–A–H), enhance the compactness of the transition zone of the reinforced interface, and improve the mechanical properties and durability of materials [22,23]. Du et al. [24] adopted a steel-slag-to-cement ratio of 3:7 and added 4% silica fume and 1% desulfurized gypsum; they observed increased contents of C–S–H and ettringite (AFt) in cementitious materials and the 28 d strength of the slurry was increased by 32.4%. Miah et al. [25] claimed that the alkaline environment provided by the hydration of highly alkaline steel slag can promote the release of glass in fly ash and the cementitious activity of C_2_S and C_3_S in steel slag powder. Zhao et al. [26] used X-ray diffraction (XRD) and gas chromatography analysis to study the effect of the specific surface area of steel slag on the activity of cementitious materials. The results showed that the fine grinding of steel slag obviously increased the hydration activity of materials; moreover, for a specific surface area of 450 m^2^/kg, the strength was significantly enhanced. With the help of the Krstulovic–Dabic kinetic model, Wu et al. [27] found that as the steel slag content increases, both the hydration heat release rate of the steel slag cementitious system and the compactness of the slurry structure decrease. Hu et al. [28] found that the steel slag–silica fume complex can yield high-impermeability concrete under the condition of a high water-to-binder ratio. At present, in the field of building materials developed from bulk industrial waste, the control variable method is often used in the preparation of steel slag cement-based gel slurry (SCGS) to study the influence of a single factor on the mechanical properties of materials; however, this method cannot reflect the influence of the interaction between various factors. There are two problems associated with the optimal preparation parameters: the lack of comprehensiveness of test conclusions and the low model accuracy.

To remedy the abovementioned shortcomings, we introduced the response surface method [29,30,31] into the whole process of SCGS preparation and research and systematically designed the experimental protocol. Subsequently, we analyzed the results and explored the influence of the interaction of various factors on the strength of SCGS. Afterward, we established the response surface regression model and optimized the parameters to achieve the optimal strength. Further, we characterized the material microstructure using scanning electron microscopy (SEM) and XRD and identified the strength formation mechanism from a microscopic perspective. Thus, the findings of this study could provide theoretical support for the application of industrial waste to SCGS.

## 2. Materials and Methods

### 2.1. Raw Materials

The cement used in the test was an ordinary Portland cement of strength class 42.5 from Horse Racing, which complies with the Chinese national standard (GB 175-2007). The steel slag (SS) was converter steel slag provided by a steel plant in Shizuishan City, Ningxia Province, China; the moisture content was 0.27% after half a year, the average particle size reached 35.18 μm after drying and grinding in a YXQM-20L planetary mill for 50 min, and the Burt’s specific surface area was 439.2 m^2^/kg. The silica fume (SF) was provided by Ningxia Ketong New Material Co., Ltd. (Shizuishan, China); the particle size was less than 1 μm for more than 81%, the average particle size was 0.1–0.4 μm, the Burt’s specific surface area was 28 m^2^/g, and the 28 d activity index was 103. The fly ash (FA) was provided by Ningxia Ketong New Material Co., Ltd.; the average particle size was 6 μm, the density was 2.9 g/cm^3^, and the fineness modulus of fine aggregate was 2.9 complying with the Chinese national standard (GSB08—1337). The water-reducing agent was a high-efficiency polycarboxylic acid (PA) powder and the water used was the tap water of the laboratory. The chemical composition of raw materials was analyzed via X-ray fluorescence spectrometry and the results are listed in Table 1.

**Figure 1:**
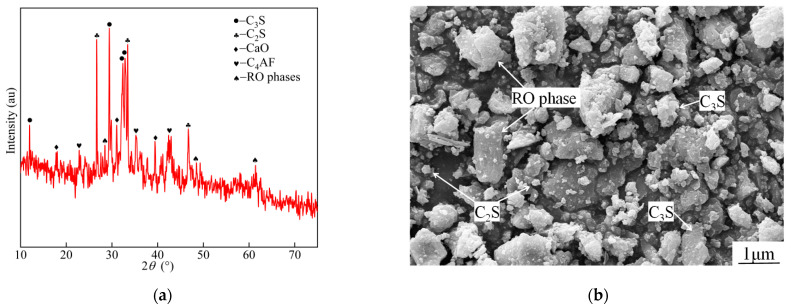
Steel slag physical properties analysis: (**a**) XRD and (**b**) SEM.

The XRD pattern and SEM diagram of steel slag are shown in Figure 1. As shown in Figure 1a, the main mineral phases of steel slag were found to be C_2_S, C_3_S, C_4_AF, and RO (solid solution composed of divalent metal oxides). As shown in Figure 1b, the steel slag particles exhibited irregular polyhedra with sharp angles and a denser but slightly uneven texture on the surface. The morphologies of C_2_S, C_3_S, and RO phases were round granular leaf-like, hexagonal plate-like, and irregular, respectively; these mineral phases were about 0.5~1.7 μm long and 0.2~0.9 μm wide. Based on the evaluation formula of slag alkalinity *M* proposed by Mason [32], the slag alkalinity was calculated to be 2.59, which indicated a highly alkaline slag. The calculation formula is as follows:

(1) $$M=\frac{\omega (CaO)}{\omega (P_{2}O_{5})+\omega (SiO_{2})}$$

where ω represents the mass fraction.

### 2.2. Box–Behnken Experimental Design

The compressive and flexural strengths of the SCGS at 3 and 28 d of age were used as the response values with the silica fume substitution rate, fly ash substitution rate, and curing temperature as the influencing factors to reveal the effect law of the single factors and interactions of independent variables on the strength of the SCGS. The Box–Behnken experimental design was applied in Design-Expert software, where three factors and three levels were selected. The codes and levels of each factor are listed in Table 2. The second-order polynomial model expressions that were used to predict the optimal preparation parameters of the SCGS were as follows:

(2) $$Y_{i}=b_{0}+\sum_{i=1}^{k}b_{i}X_{i}+\sum_{i=1}^{k}b_{ii}X_{i}^{2}+\sum_{i=1}^{k}\sum_{j>1}^{k}b_{ij}X_{i}X_{j}+\varepsilon$$

where *Y_i_* is the predicted response of compressive strength and flexural strength at each age; *b*_0_ is a constant; *X_i_* and *X_j_* are the coded values of the independent process variables; *k* is the number of independent variables, i.e., *k = 3*; *b_i_* is the linear coefficient; *b_ii_* is the quadratic coefficient; *b_ij_* is the interaction coefficient; and *ε* is the random error of the prediction.

### 2.3. Test Methods

#### 2.3.1. Macroscopic Test Method

According to [33] and the test results of the preliminary trial, the steel slag dose was 15%, the water-to-cement ratio was 0.4, and the water-reducing agent dose was 0.14% of the amount of cementitious material. The cementitious materials were weighed according to the Box–Behnken test design. They were first mixed dry in a JJ-5 planetary type cementitious sand mixer for 120 s. Afterward, the water-reducing agent was dissolved in the required water and poured into the mixer for 120 s. Finally, the standard sand was poured into the mixer and mixed for 180 s. The cementitious materials were poured into a 40 × 40 × 160 mm mold and vibrated to test the compressive strength and flexural strength. The test was carried out in a standard curing box at a temperature of 20 ± 2 ℃ and relative humidity of 95% or more for 24 h. After demolding, each mold was placed in a curing box at the specified curing temperature (20, 50, or 80 ℃) and relative humidity (95% or more) until 3 or 28 d of age.

The KZL-5000 cement electric flexural testing machine was used to perform flexural strength testing and the YAW-300D electronic pressure testing machine was used to perform compressive strength testing; the vertical loading speed for testing was 0.5 kN/s. Three parallel specimens were prepared for each mixing ratio.

#### 2.3.2. Microscopic Test Method

According to the optimal mixing ratio determined by the optimized parameter plate in Design-Expert, the cementitious materials were weighed and the pure slurry of the SCGS optimized group was prepared with a water-to-cement ratio of 0.4. The pure slurry of the reference group was prepared with 15% steel slag, 85% cement, and a 0.4 water-to-cement ratio. The hydration mechanism was studied by pouring the cementitious materials into a 40 × 40 × 40 mm mold for vibration molding. The optimized group was cured at a temperature set according to the optimal preparation parameters and relative humidity greater than 95%, whereas the reference group was cured under standard conditions. Each sample was taken out after 3 or 28 d of hydration for compressive strength testing. After the compressive strength test, the central broken block was taken as the microscopic test sample and placed in anhydrous ethanol to terminate the hydration. The samples were dried in a vacuum drying oven at 60 ℃ to constant weight before the XRD and SEM tests. Subsequently, they were pressed into powder with an agate mortar and then subjected to XRD analysis, and another flat sample was selected for SEM analysis after gold plating. XRD analysis was performed using a D8 Advance X-ray diffractometer from Bruker, Germany, with Cu as the anode target and a scanning range of 10° to 73° at intervals of 0.02°. The SEM analysis was carried out using a Zeiss EVO 18 scanning electron microscope.

## 3. Results and Discussion

### 3.1. Experimental Results

The results based on the Box–Behnken experimental design and response values are listed in Table 3.

### 3.2. RSM Analysis of Variance (ANOVA) and Reliability Assessment

The results of the response values were analyzed by performing a regression analysis with the quadratic RSM using Design-Expert software to establish the regression models of compressive strength and flexural strength of the SCGS at 3 and 28 d of age with silica fume replacement rate, fly ash replacement rate, and curing temperature. The resulting multivariate quadratic response surface regression models are expressed as follows:

*Y*_1_ = 36.857 − 0.93092*X*_1_ − 0.281617*X*_2_ − 0.101403*X*_3_ − 0.0783*X*_1_*X*_2_ + 0.0246*X*_1_*X*_3_ + 0.0074*X*_2_*X*_3_ + 0.10038*X*_1_^2^ + 0.00516*X*_2_^2^ − 0.001162*X*_3_^2^, (3)

*Y*_2_ = 30.605 + 3.08554*X*_1_ − 0.07645*X*_2_ + 0.366*X*_3_ + 0.0825*X*_1_*X*_2_ + 0.008042*X*_1_*X*_3_ + 0.00735*X*_2_*X*_3_ + 0.39275*X*_1_^2^ − 0.00944*X*_2_^2^ − 0.004098*X*_3_^2^, (4)

*Y*_3_ = 7.774 − 0.91063*X*_1_ + 0.15925*X*_2_ + 0.004361*X*_3_ + 0.04075*X*_1_*X*_2_ + 0.01175*X*_1_*X*_3_ + 0.0008*X*_2_*X*_3_ + 0.014687*X*_1_^2^ − 0.01465*X*_2_^2^ − 0.000649*X*_3_^2^, and (5)

*Y*_4_ = 6.531 − 0.28304*X*_1_ + 0.08495*X*_2_ + 0.04444*X*_3_ + 0.03925*X*_1_*X*_2_ + 0.00233*X*_1_*X*_3_ + 0.0042*X*_2_*X*_3_ + 0.004312*X*_1_^2^ − 0.01751*X*_2_^2^ − 0.00075*X*_3_^2^, (6)

where *Y*_1_ and *Y*_2_ are the 3 and 28 d compressive strengths, respectively, and *Y*_3_ and *Y*_4_ are the 3 and 28 d flexural strengths, respectively.

ANOVA was performed on the established response surface regression models *Y*_1_ and *Y*_2_, with the results presented in Table 4.

The significances of the linear, quadratic, and interaction effects of the influencing factors in the response surface regression model equation were determined by both the *F*- and *P*-values of the model. The larger the *F*-value is and the smaller the *P*-value is, the more significant the influence effect is. When *P* < 0.05, the relationship between the response surface *Y_i_* and the regression model and the relationship between each effect term and the response surface *Yi* are significant, otherwise the opposite is true. The reliability of the regression model fit is assessed using the correlation coefficient *R*^2^, which represents the degree of difference between the measured and predicted values; the closer its value is to 1, the higher the reliability of the fit is.

As shown in Table 4, model *Y*_1_ had an *F*-value of 20.88 with a *P*-value of 0.0003, and model *Y*_2_ had an *F*-value of 26.26 with a *P*-value of 0.0001. The fact that the *P*-values were less than 0.05 indicated that the regression effects of models *Y*_1_ and *Y*_2_ were highly significant. The *P*-values corresponding to *X*_1_, *X*_2_, and *X*_3_ in model *Y*_1_ were 0.0073, 0.009, and less than 0.0001, respectively. The *P*-values corresponding to *X*_1_, *X*_2_, and *X*_3_ in model *Y*_2_ were 0.0002, 0.0001, and 0.0006, respectively. The fact that the *P*-values were less than 0.05 indicated that every single factor had a significant effect on the compressive strength of the SCGS at 3 and 28 d of age. Because *P*(*X*_2_) > *P*(*X*_1_) > *P*(*X*_3_) in model *Y*_1_, the single-factor significance was *X*_3_ > *X*_1_ > *X_2_*. Because *P*(*X*_3_) > *P*(*X*_1_) > *P*(*X*_2_) in model *Y*_2_, the single-factor significance was *X*_2_ > *X*_1_ > *X*_3_. The *P*-values of the interaction terms *X*_1_*X*_2_, *X*_1_*X*_3_, and *X*_2_*X*_3_ in model *Y*_1_ were 0.0130, 0.0004, and 0.0022, respectively, i.e., less than 0.05, indicating that the interaction of the three factors had a significant effect on the compressive strength of the SCGS at 3 d of age. The *P*-values of the interaction terms *X*_1_*X*_2_, *X*_1_*X*_3_, and *X*_2_*X*_3_ in model *Y*_2_ were 0.1074, 0.3161, and 0.0430, respectively. Except for *P*(*X*_2_*X*_3_), the *P*-values were all greater than 0.05; hence, except for the interaction of *X*_2_*X*_3_, the interaction of the factors had no significant effect on the compressive strength of the SCGS at 28 d of age.

**Figure 2:**
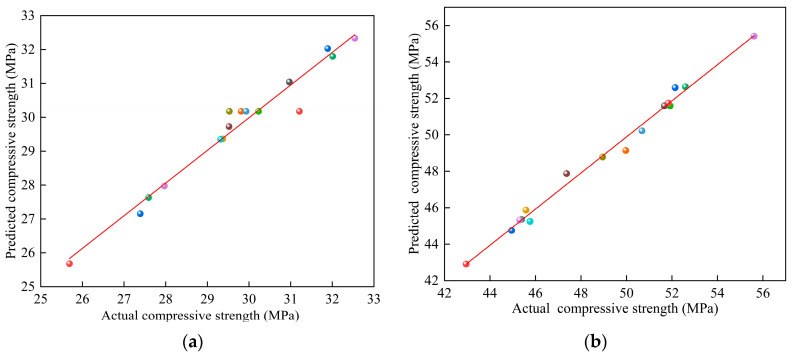
The relationship between the experimental values and predicted values: (**a**) *Y*_1_ and (**b**) *Y*_2_.

The correlation coefficient *R*^2^ = 0.9642, modified correlation coefficient *R*_Adj_^2^ = 0.9179, and predicted correlation coefficient *R*_pred_^2^ = 0.9102 for model *Y*_1_, as well as the correlation coefficient *R*^2^ = 0.9712, modified correlation coefficient *R*_Adj_^2^ = 0.9343, and predicted correlation coefficient *R*_pred_^2^ = 0.9161 for model *Y*_2_, were all close to 1; this confirmed that models *Y*_1_ and *Y*_2_ had high accuracy and reliability regarding the prediction. The x–y scatter plots were drawn with the measured and predicted values of models *Y*_1_ and *Y*_2_ as the horizontal and vertical coordinates, respectively, as shown in Figure 2. As shown in Figure 2, the scatter points were uniformly distributed around the y = x line; thus, the measured and predicted values were in high agreement, indicating that the model was fitted with high accuracy and corroborated the results of the response surface regression model ANOVA.

### 3.3. Analysis of Response Surface Influencing Factors

#### 3.3.1. Influence and Interaction of Compressive Strength of the SCGS at 3 d of Age

**Figure 3:**
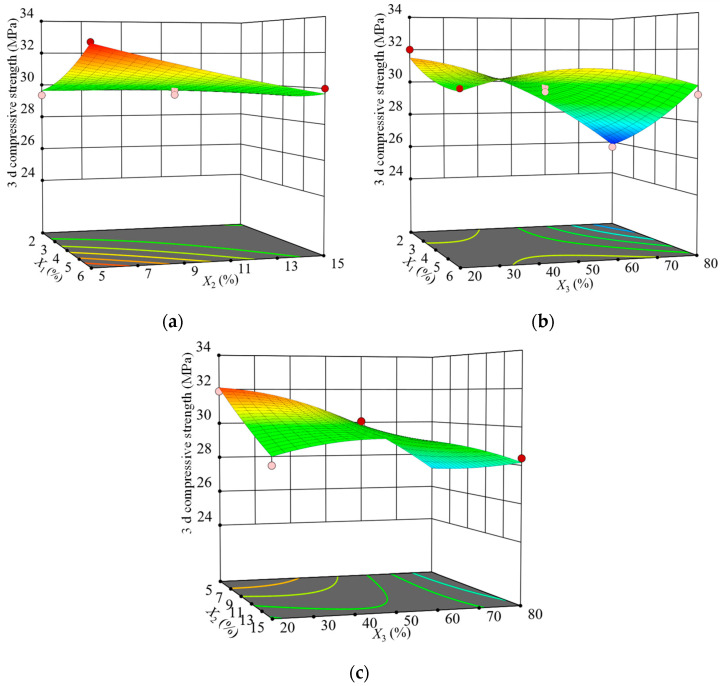
Influences of the interactions of various factors on the 3 d compressive strength: (**a**) *X*_1_ and *X*_2_, (**b**) *X*_1_ and *X*_3_, and (**c**) *X*_2_ and *X*_3_.

To intuitively study the effect of the interaction of silica fume replacement rate, fly ash replacement rate, and curing temperature on the strength, the three-dimensional (3D) surface plot of the the SCGS compressive strength at 3 d of age at the three-factor level was drawn according to the regression model. The results are shown in Figure 3.

Figure 3a depicts the effect of the interaction between the silica fume and fly ash on the SCGS’s strength at a fixed maintenance temperature of 50 ℃. The 3D surface plot was obviously twisted, indicating that the interaction between silica fume and fly ash had a significant effect on the early strength. When the fly ash substitution rate was low, the strength increased with the silica fume substitution rate and the curve increased significantly, indicating that the silica fume promoted the early strength. Further, the hydration of cement and steel slag in the matrix ensures an alkaline environment, and silica fume, which is rich in highly reactive SiO_2_, is constantly in contact with the solution at the early stage of hydration. Consequently, a Si-rich and Ca-poor gel adhesive layer is formed on the surface of the particles. As the hydration reaction proceeds, the layer gradually dissolves and reacts with the hydration product Ca(OH)_2_ crystals in a volcanic ash reaction to generate C–S–H gels. This limits the Ca(OH)_2_ inhibitory effect on growth and improves the interface between steel slag micronized fly ash and the C–S–H gel transition zone. Conversely, fly ash has a microaggregate effect and plays a physical filling role, together with silica fume and the unhydrated steel slag micropowder, which do not participate in the volcanic ash reaction to optimize the pore structure, leading to a large increase in the early strength.

According to Figure 3a, the strength decreased as the fly ash substitution rate increased, which indicated that the early volcanic ash activity of fly ash was low after replacing part of the cement and the hydration of steel slag micronized powder in the SCGS system itself was slow. Thus, the prehydration reaction had a limited ability to provide OH^−^, which made it difficult to cause the fly ash reticulation structure to break, the volcanic ash effect occurred slowly, and the consumption rate of Ca(OH)_2_ crystals was slow, resulting in a lower strength in the early stage. In addition, the microaggregate effect of fly ash fills between cement and steel slag powder, which, to some extent, hinders the contact of water molecules with cement and steel slag powder, and the slow hydration process of cementitious materials lead to the slow hardening of the slurry.

Figure 3b shows the effect of the interaction between silica fume and maintenance temperature on the SCGS’s strength at a fixed fly ash substitution rate of 15%. The twist degree of the 3D surface plot was more obvious, indicating that the interaction between silica fume and maintenance temperature had a highly significant effect on the early strength, which is consistent with the ANOVA results. At a higher silica fume substitution rate, as the curing temperature increased, the strength first increased, and then decreased, with its peak occurring around 50 ℃. The reason was that, when warming to a certain temperature, the average molecular chain length of the cement hydration product C–S–H gel grows significantly compared to that at 20 ℃. Moreover, warming accelerates the excitation of fly ash and silica fume volcanic ash activity, consumes Ca(OH)_2_ crystals, and provides space for the growth of C–S–H chains [34], which effectively fills the pores of the interfacial transition zone and increases the SCGS’s strength. In addition, warming accelerates the early hydration rate of cement, increases the production of hydration products, and increases the SCGS’s strength. However, the strength decreased when the temperature increased to 80 ℃. This may have been due to the fact that, at the late stage of hydration, the paste was solid, the hydration reaction slowed down, and the temperature was no longer a key factor for strength. At lower temperatures, the strength increase did not change much with the silica fume replacement rate. At higher temperatures, the SCGS’s strength increased linearly with the silica fume substitution rate.

Figure 3c shows the effect of the interaction between fly ash and maintenance temperature on the SCGS’s strength at a fixed silica fume replacement rate of 4%. Obviously, the 3D surface plot twisting trend was similar to that in Figure 3b, but the degree of twisting was slightly lower than that in Figure 3b, indicating that the interaction of the fly ash and maintenance temperature had a significant effect on the early strength, which was consistent with the ANOVA results. At lower temperatures, the strength decreased linearly as the fly ash replacement rate increased. However, fly ash mainly played the role of a microaggregate in the early stage and contributed less to the strength. Conversely, the cement replacement with fly ash reduced the number of gels generated by the hydration reaction, such as high-density C–S–H and C–A–H, and reduced the strength. At higher temperatures, the strength increased linearly with the fly ash substitution rate, but the increase was not significant.

#### 3.3.2. Effect and Interaction of Compressive Strength of the SCGS at 28 d of Age

**Figure 4:**
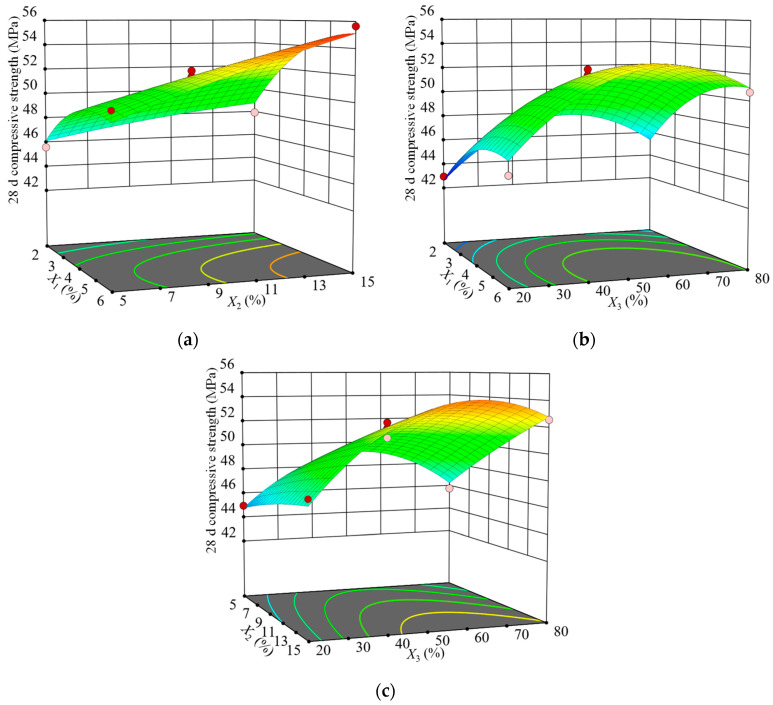
Influences of the interactions of various factors on the 28 d compressive strength: (**a**) *X*_1_ and *X*_2_, (**b**) *X*_1_ and *X*_3_, and (**c**) *X*_2_ and *X*_3_.

The 3D surface plots of the compressive strength of the SCGS at 28 d of age at the three-factor level were drawn according to the regression model. The results are shown in Figure 4.

Figure 4a shows the effect of the interaction between silica fume and fly ash on the SCGS’s strength at a fixed maintenance temperature of 50 °C. The 3D surface plot was not obviously twisted, indicating that the interaction between the silica fume and fly ash had no significant effect on the later strength, which was consistent with the ANOVA results. At lower fly ash replacement rates, as the silica fume replacement rate increased, the strength first increased and then leveled off, but its growth was small, indicating that the silica fume contribution to the later strength was weaker than the earlier one. At higher fly ash replacement rates, the superposition effect of silica fume and fly ash caused the strength to increase rapidly. The strength increased with the fly ash replacement rate, which indicated that the fly ash contributed to the later strength growth. The steel slag and cement hydration process and the high alkalinity of the liquid phase contribute to the depolymerization of [SiO_4_] and [AlO_4_] tetrahedra in the fly ash and the secondary hydration reaction with Ca(OH)_2_, which generates C–S–H and C–A–H gels to fill the pores between the primary hydration products of cement [34]; this makes the slurry denser and thus increases the strength.

Figure 4b shows the effect of the interaction between the silica fume and curing temperature on the SCGS’s strength at a fixed fly ash substitution rate of 15%. The surface plot had a downward opening and convex shape and included great values for optimal analysis. However, the interaction between the silica fume and curing temperature had no significant effect on the later strength, which was consistent with the ANOVA results. In the range of the silica fume substitution rate, as the curing temperature increased, the strength first increased and then decreased, with its peak occurring around 50 °C, and the reason for this was consistent with the abovementioned effect of temperature on strength. In the range of the curing temperature, as the silica fume substitution rate increased, the strength first increased and then decreased, with its peak occurring around a 4% rate. The reason was that, although the volcanic ash effect and filling effect of silica fume made the internal structure of the slurry denser and the strength increased, the amount of silica fume was too large for the later strength growth.

Figure 4c shows the effect of the interaction between fly ash and maintenance temperature on the SCGS’s strength at a fixed silica fume replacement rate of 4%. The 3D surface was significantly twisted, indicating that the interaction of the fly ash and curing temperature had a significant effect on the later strength, which was consistent with the ANOVA results. At higher temperatures, the strength increased significantly with the fly ash substitution rate because such temperatures excited the volcanic ash activity of fly ash, and more fly ash reacted with Ca(OH)_2_, thereby generating gels, such as C–S–H and AFt. Consequently, the highly crystalline Ca(OH)_2_ was consumed, making the pore structure denser and the strength higher. Regardless of the value of the fly ash substitution rate, as the temperature increased, the strength first increased and then decreased, with its peak occurring around 55 ℃.

### 3.4. Parameter Optimization and Validation

Based on the compressive strength optimization of the SCGS mortar at 3 and 28 d of age using Design-Expert, the strength optimization scheme of the SCGS mortar was obtained by combining the interaction analysis of various factors. To verify the results of the response surface optimization, three sets of parallel tests were conducted to determine the mechanical properties of the SCGS mortar at each age, where the test results are listed in Table 5.

As shown in Table 5, the error between the predicted and measured values of the compressive strengths at 3 and 28 d of age were 2.51% and 3.35%, respectively. The errors were all less than 5%, showing that the optimal preparation parameters obtained using the response surface method were of high precision in terms of both practice and theory, further verifying the rationality of the regression model and supporting the promotion of the SCGS mortar’s strength value and reference significance.

## 4. Microscopic Mechanism of Action Analysis

### 4.1. XRD Analysis

**Figure 5:**
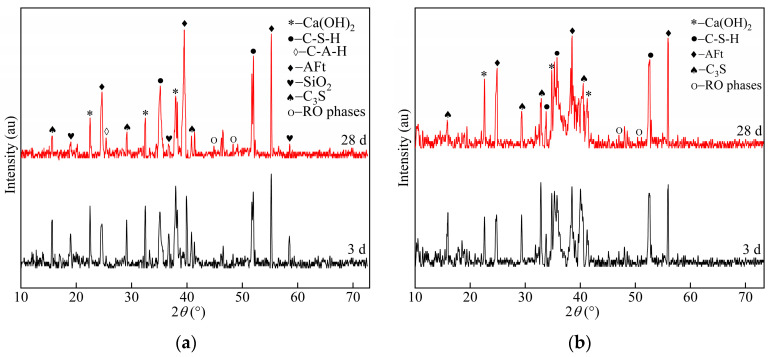
XRD patterns at 3 and 28 d of age according to the (**a**) optimized group samples and (**b**) reference group samples.

The pure paste specimens of the SCGS optimized group at each age were selected for XRD tests. The content changes of hydration products were qualitatively analyzed according to age for both groups and were compared with the pure paste specimens of the reference group at each age. The results are shown in Figure 5.

Figure 5a shows the XRD patterns of the optimized specimens at 3 and 28 d of age. The hydration products were dominated by calcium alumina (AFt), calcium silicate hydrate (C–S–H), calcium hydroxide (CH), silica (SiO_2_), RO phase, unhydrated dicalcium silicate (C_2_S), and other mineral phases. RO phases, as inert minerals in the steel slag, played a filling role in the system and did not participate in the hydration process; hence, their diffraction peaks remained stable with age.

At 3 d of age, the specimens of the optimized group showed obvious peak packets at diffraction angles within the range 33°–38°, and the diffraction peak widths of the AFt and C–S–H phases were wider; in addition, the peak values and widths of the AFt and C–S–H diffraction peaks increased with the curing time, indicating that the production of AFt and C–S–H in the hardened slurry was increasing. At a diffraction angle near 18°, i.e., the SiO_2_ characteristic peak, which mainly originated from the silica fume and fly ash, the diffraction peaks of C_3_S and Ca(OH)_2_ in 3 d were higher, but the peaks and widths of C_3_S and Ca(OH)_2_ gradually became weaker with the progress of the hydration reaction. The above results indicated that C_3_S in cement and steel slag had high hydration and continuously generated Ca(OH)_2__._ However, the XRD pattern reflected the fact that the peak of Ca(OH)_2_ decreased. This occurred because the secondary hydration of SiO_2_ accelerated the Ca^2+^ absorption, and Ca(OH)_2_ consumption was greater than its generation; the more rapid the reaction was, the more the paste’s early strength increased. In addition, the hydration of C_3_S in steel slag generated Ca(OH)_2_, which was absorbed by fly ash as the hydration process proceeded because of its relatively low crystallinity; thus, the diffraction peak corresponding to Ca(OH)_2_ was weak in the XRD pattern.

The weak C–A–H diffraction peaks of the optimized specimens at 28 d of age indicated that, under a high temperature and alkaline environment, the fly ash structure gradually dissociated and a certain number of Si and Al atoms dissolved and reacted with Ca^2+^ in the solution to form C–S–H and C–A–H gels, respectively. However, the intensity of the C–A–H diffraction peak was weak because the liquid phase of SO_4_^2−^ and C–A–H’s structural instability gradually increased the amount of AFt. As the age increased, the hydration products C–S–H and AFt of the hardened slurry continued to increase, which ensured the increasing strength of the slurry.

Figure 5b shows the hydration products of the specimens in the reference group. The XRD patterns show the diffraction peaks of AFt, Ca(OH)_2_, and C–S–H gels at 3 and 28 d of hydration in the reference group, where the peaks and widths of the diffraction peaks of AFt, Ca(OH)_2_, and C–S–H gels increased gradually with the curing age. The C_3_S diffraction broad peak, which mainly originated from cement and steel slag, appeared near 32° and weakened in the late hydration period. The diffraction peak of Ca(OH)_2_ at 28 d of age was obviously stronger than that of the optimized group because the late hydration of C_2_S and C_3_S mineral activity in steel slag did not consume Ca(OH)_2_ but hydrated to produce Ca(OH)_2_ and C–S–H gels. Furthermore, the cement Ca(OH)_2_ was continuously produced in the late stage of hydration, which gradually strengthened the diffraction peaks. The precipitation of large Ca(OH)_2_ crystals in the interfacial transition zone of the steel slag cement system caused the matrix strength degradation owing to the large porosity.

### 4.2. SEM Analysis

**Figure 6:**
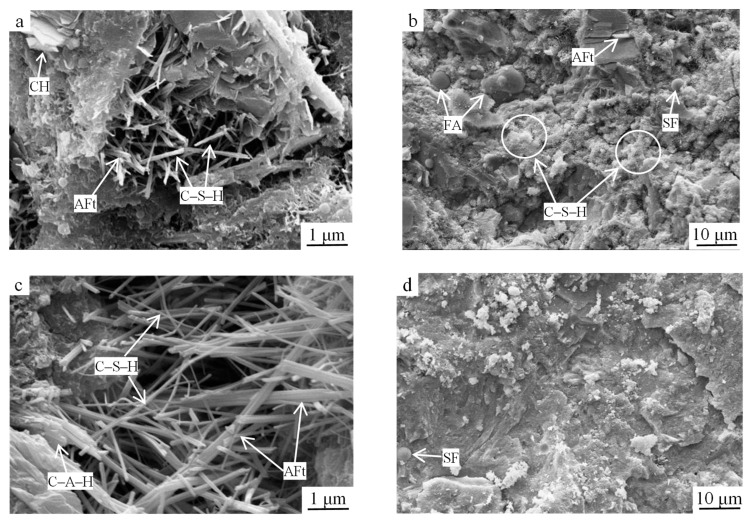
SEM images of the optimized group samples at (**a**,**b**) 3 d of age and (**c**,**d**) 28 d of age. SF: silica fume, FA: fly ash.

**Figure 7:**
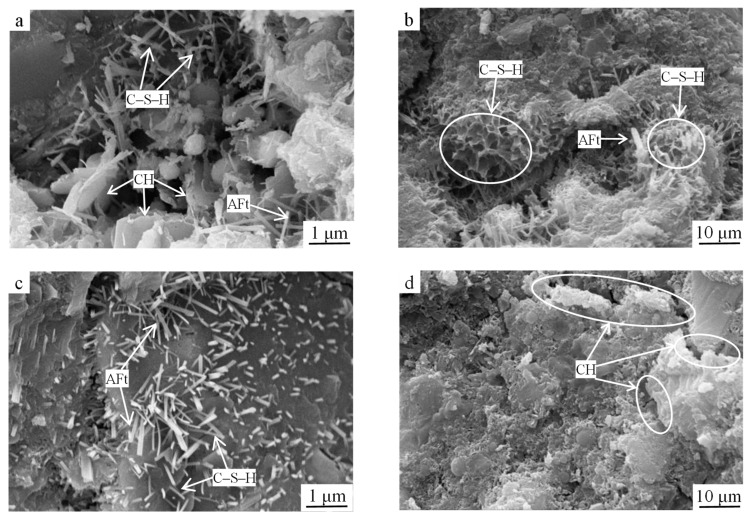
SEM images of the reference group samples at (**a**,**b**) 3 d of age and (**c**,**d**) 28 d of age.

To further investigate the SCGS’s strength development law, the microscopic morphology inside the hardened slurry was observed via SEM. Figure 6 shows the SEM results of the optimized group, and Figure 7 shows the SEM results of the reference group.

At 3 d of age, the observed hydration products were flocculent fibrous C–S–H gels, rodlike AFt crystals, hexagonal platelike Ca(OH)_2_, unhydrated cement particles, and steel slag micronized powder. The rodlike AFt crystals were interspersed with fibrous C–S–H and interwoven to fill the pores between the unhydrated cement and steel slag particles, and the mesh structure of the hardened slurry was formed (Figure 6a). The spherical fly ash particles with smooth surfaces did not participate in the hydration process at the early stage but played the role of aggregate filling; they were wrapped by the deposited hydration products one by one and were tightly bonded to the surrounding slurry (Figure 6b). Thus, the early strength of the slurry reached 31.78 MPa compared to the 27.63 MPa of the reference group, which was an increase of 15.02%.

At 28 d of age, with the continuous development of the gel system, the number of Ca(OH)_2_ crystals decreased, and the hydration products were dominated by C–S–H gels and rodlike Aft, which both grew significantly in size and had a higher degree of crystallization (Figure 6c). On the one hand, this was because the Si–O and Al–O bonds in the fly ash structure were mainly in the form of a 3D network linked by [SiO_4_] and [AlO_4_] tetrahedra, and the hydration of cement and steel slag increased the pH of the liquid phase, prompting the rapid depolymerization of the low-strength O–Si–O and Si–O–Al chemical bonds [15]. At that time, the solution contained a large amount of Ca^2+^, OH^−^, SO_4_^2^^−^, H_3_SiO_4_^−^, and H_3_AlO_4_^2−^ ions, providing a material basis for the formation of AFt. On the other hand, the fly ash gradually increased its participation in the hydration reaction, and secondary hydration occurred, producing Ca(OH)_2_ to generate C–S–H and C–A–H gels. As the hydration process proceeded, SO_4_^2−^ reacted with C–A–H gel under the action of Ca^2+^ to form AFt crystals. Under certain temperature and highly alkaline conditions, the steel slag surface was severely eroded and wrapped by flocculent C–S–H gel and the hydration of steel slag gradually increased; thus, C–S–H gel and Ca(OH)_2_ were generated, and the concentrations of Ca and Si increased, leading to thicker AFt crystals, C–S–H gelling, and further AFt generation, which formed a tight mesh structure. Subsequently, silica fume particles that were not involved in the pozzolanic reaction filled the slurry and bonded with the hydration products into a whole, forming a dense microstructure (Figure 6d). As a result, the 28 d strength of the slurry reached 53.94 MPa compared to the 44.85 MPa of the reference group, which was an increase of 11.96%.

As shown in Figure 7a, at 3 d of age, the contents of C–S–H gel and AFt of the reference group specimens were significantly reduced compared to those in Figure 6a. One reason for this was that the system was subjected to only one hydration cycle, generating relatively few hydration products. Another reason was that the earlier cement hydration generated limited Ca(OH)_2_, and the insufficient alkalinity in the liquid phase resulted in the low hydration rate of steel slag. Steel slag on the RO phase particles’ surfaces attached some of the gel and smaller steel slag particles, affecting the strength of the particles and surrounding hydration products’ association; meanwhile, more internal macropores and connected pores were created since the hydration-generated C–S–H gelation was mainly honeycomb-structured, which was loose (Figure 7b) and thus not conducive to the growth of the early strength of the specimen, and hence the reference group strength was low. With the extension of the hydration time, the hydration products generated in the system at 28 d increased significantly, perhaps because the alkaline environment generated by the hydration of the cement excited the steel slag activity and started the hydration reaction and the contents of the C–S–H gel and AFt in the system increased; however, compared to the optimized group at the same age, the contents of the C–S–H gel and rodlike AFt were low and loosely distributed (Figure 7c). It is noteworthy that the lamellar Ca(OH)_2_ crystals in the reference group were enriched in the interfacial transition zone and had large crystalline grains (Figure 7d) and their loose interlayer stacking connections weakened the bond strength in the interfacial transition zone, which was the weak link of the hardened slurry; thus, the strength of the reference group was lower than that of the optimized group.

## 5. Conclusions

The effects of silica fume substitution rate, fly ash substitution rate, and curing temperature on the compressive strength of the SCGS at 3 and 28 d of age were investigated via the RSM. The hydration products and microscopic morphology of the pure slurry were analyzed based on the optimal preparation parameters obtained using Design-Expert, and the following conclusions were drawn.

(1) The regression models for the compressive strength of the SCGS at 3 and 28 d of age were established via response surface regression analysis. From the ANOVA and 3D surface plots, it was found that the interaction between the three factors had a significant effect on the compressive strength of the SCGS at 3 d of age and the interaction of fly ash and maintenance temperature had a significant effect on the compressive strength of the SCGS at 28 d of age.

(2) The sensitivity ranking of the single factor response to the compressive strength of the SCGS at 3 d of age was curing temperature > silica fume > fly ash; the effect of curing temperature and silica fume on the early strength of the SCGS was significant within a certain range, whereas the contribution of fly ash was limited. The sensitivity ranking of the single factor response to the compressive strength of the SCGS at 28 d of age was fly ash > silica fume > curing temperature; the effect of fly ash on the later strength of the slurry was significant.

(3) The optimal preparation parameters for the compressive strength of the SCGS at 3 d of age were obtained from the prediction model established via response surface analysis. These were as follows: 5.8% silica fume substitution rate, 5.2% fly ash substitution rate, and 43.3 ℃ curing temperature. The optimal preparation parameters for the compressive strength of the SCGS at 28 d of age were as follows: 4.2% silica fume substitution rate, 15% fly ash substitution rate, and 59.2 ℃ curing temperature. The actual compressive strengths at 3 and 28 d of age were 31.78 and 53.94 MPa, respectively, which were close to the predicted values (32.59 and 55.81 MPa, respectively), demonstrating that the RSM-optimized SCGS strengths were accurate and reliable.

(4) The hydration reaction rate of SiO_2_ in the silica fume and the physical filling effect of the inert components of the fly ash and steel slag under the optimal parameters were the key factors for the early strength of the material. The continuous hydration of C_3_S in the steel slag and the continuous excitation of the volcanic ash properties of the fly ash were important factors for the later strength. The hydration of active SiO_2_ and Al_2_O_3_ in the fly ash consumed Ca(OH)_2_, which, in turn, was significantly reduced in content and crystal size; this generated a thick fibrous S–C–H gel and rod-like Aft, which formed a dense network structure, improving the early strength of the cementitious system. The precipitation of large Ca(OH)_2_ crystalline particles in the interfacial transition zone of the steel slag cement system gradually deteriorated the matrix strength because of the large porosity between the layers.

## Figures and Tables

**Table 1:** Main chemical compositions of the raw materials (%).

Raw Materials	Mass Fraction (%)									
	SiO_2_	Al_2_O_3_	Fe_2_O_3_	CaO	MgO	K_2_O	Na_2_O	TiO_2_	SO_3_	P_2_O_5_
Cement	16.41	4.14	4.09	65.32	1.48	0.13	0.28	—	2.20	0.18
Silica fume	95.96	0.29	0.02	0.12	0.31	0.03	—	0.06	—	—
Fly ash	45.34	31.93	7.56	4.88	1.21	2.39	0.81	1.68	0.69	—
Steel slag	15.26	3.12	20.98	43.69	4.36	0.02	0.13	1.85	0.39	1.61

**Table 2:** Factor codes and levels.

Influencing Factor	Code	Level		
SF substitution rate (%)	*X_1_*	2	4	6
FA substitution rate (%)	*X_2_*	5	10	15
Curing temperature (℃)	*X_3_*	20	50	80

**Table 3:** Box–Behnken experimental design and response values.

Number	Factor			Response			
	*X*_1_ (SF (%))	*X*_2_ (FA (%))	*X*_3_ (CT (℃))	Compressive Strength (MPa)		Flexural Strength (MPa)	
				3 d	28 d	3 d	28 d
T1	4	10	50	29.81	51.67	7.11	8.95
T2	4	5	80	27.59	45.76	5.91	7.68
T3	6	10	20	29.97	45.31	6.74	8.58
T4	4	10	50	30.21	51.61	7.53	9.21
T5	4	15	20	28.23	47.37	6.89	6.93
T6	4	15	80	28.39	52.59	6.73	9.71
T7	6	15	50	30.01	55.64	8.41	9.88
T8	6	10	80	29.52	50.68	7.89	9.83
T9	4	10	50	29.53	52.14	7.25	9.39
T10	6	5	50	32.54	49.97	6.79	7.83
T11	4	5	20	31.89	44.95	6.55	7.42
T12	2	10	20	32.01	42.95	7.41	7.23
T13	4	10	50	29.93	51.62	7.93	8.47
T14	2	15	50	29.97	47.95	6.72	8.64
T15	2	10	80	25.69	46.39	5.74	7.92
T16	2	5	50	29.37	45.58	6.73	8.16
T17	4	10	50	30.23	50.92	7.53	9.22

**Table 4:** Analysis of variance of response surface regression model.

Source	Sum of Squares		Freedom		Mean Square		*F*-Value		*P*-Value	
	*Y*_1_	*Y*_2_	*Y*_1_	*Y*_2_	*Y*_1_	*Y*_2_	*Y*_1_	*Y*_2_	*Y*_1_	*Y*_2_
Model	42.06	188.88	9	9	4.67	20.99	20.88	26.26	0.0003	0.0001
*X*_1_	3.13	43.58	1	1	3.13	43.95	13.96	46.76	0.0073	0.0002
*X*_2_	2.87	37.37	1	1	2.87	37.37	12.81	54.88	0.009	0.0001
*X*_3_	14.88	27.53	1	1	14.88	27.53	66.47	34.45	<0.0001	0.0006
*X*_1_ *X*_2_	2.45	2.72	1	1	2.45	2.72	10.94	3.41	0.0130	0.1074
*X*_1_ *X*_3_	8.61	0.9316	1	1	8.61	0.9316	38.48	1.17	0.0004	0.3161
*X*_2_ *X*_3_	4.97	4.86	1	1	4.97	4.86	22.22	6.08	0.0022	0.0430
*X*_1_^2^	0.6787	10.39	1	1	0.6787	10.39	3.03	13.00	0.1252	0.0087
*X*_2_^2^	0.07	0.2345	1	1	0.07	0.2345	0.313	0.2935	0.5933	0.6048
*X*_3_^2^	4.61	57.28	1	1	4.61	57.28	20.58	71.69	0.0027	<0.0001
Residual	1.57	5.59	7	7	0.23	0.7991				
Cor Total	43.63	194.48	16	16						

**Table 5:** Measured and predicted values of the SCGS’s strength.

	PO (%)	SS (%)	SF (%)	FA (%)	CT (℃)	PA (%)	Compressive Strength (MPa)	
							Predicted Value	Actual Value
3 d optimized group	74	15	5.8	5.2	43.3	0.14	32.59	31.78
28 d optimized group	65.8	15	4.2	15	59.2	0.14	55.81	53.94

## Data Availability

The data supporting the findings of this study are available from the corresponding author upon reasonable request.
